# Address

**Published:** 1834-10-01

**Authors:** 


### MEDICAL QUARTERLY REVIEW.

---

### ADDRESS.

It has long been the custom of belligerents, at the commencement of a war, to issue a manifesto, in which they declare the grounds of the contest, and the objects which it is intended to attain: and, at the opening of a literary campaign, it is usual to send forth a similar declaration, which is called a Prospectus. Again, the General publishes his dispatches, in which he enumerates the fertile provinces conquered, and the hostile armies destroyed: and we see no reason why an Editor should not, in like manner, give a Retrospective Review of his own Journal, and touch upon the valuable works analysed by the able pens of his contributors, or hint the fact, that in his pages sundry quackish sciolists have met with a tithe, or so, of the castigation they merited.

In the first place, then, we have given a number of real reviews, and not merely dry abridgments, or extracts, with a few sentences interposed to keep the peace between them. Thus, for example, the reviews of Lawrence, Mayo, Uwins, Magendie, Roupell, Wilson Philip, and Thomson, will be found to exhibit something like a general view of the subject under consideration, or, at least, will be found replete with matter not derived from the book reviewed. In the case, indeed, of some German works of great merit, which were not likely to fall into our readers' hands, we have gone further than this, and have given such an abstract, as, for ordinary purposes, may supersede the necessity of consulting the original. As examples of these epitomizing reviews, we may cite those of Beck *upon Bronchocele*, and Kramer *on Chronic Deafness.*

We promised, too, in our Prospectus, that even the smallest features of current medical literature should not escape that

Oct. 1834.

faithful chronicler, the Medical Quarterly Review; and in this point also we flatter ourselves that we have amply redeemed our pledges. Without the formality of a regular list, we have given something like a *catalogue raisonnée* of new books, good, bad, and indifferent: and if the latter classes have been more numerous than we could have wished, we hope that we shall not be blamed for having announced the fact with the most Rhadamanthine impartiality. But we are sure that we shall not be blamed; for, if there is one quality which above all others is universally appreciated, it is honesty; and, whatever faults may be found or fancied in our 1000 pages, we are quite certain that partiality and prejudice are not among the number. Some well-meaning persons, indeed, accustomed to the honied phrases of courtly criticism, may perceive nothing but harshness in the voice of truth; but we would ask these soft and milky readers to inquire what has been the fate of the books which we have condemned, and, if our censures have not been confirmed by the trunk-maker and the pastrycook, we will readily confess that our judgment has been too severe; but, if these abortive attempts have already sunk into oblivion, we must still continue our course, and, in the language of Aristophanes, "call a spade a spade, and Cleon a knave."

Our Original Communications have been more numerous than we expected, and, did we not exercise the most fastidious judgment in the selection, they might be far more numerous; but in this single point we confess that we lean rather towards rigour than indulgence; and contributions authenticated by the most respectable names have been rejected, when deficient in instruction or novelty.

In our Collectanea we have endeavoured to avoid the Scylla as well as the Charybdis of Journalists: we have neither confined ourselves to three or four meagre quotations, nor have given a chaos of extracts, through which no human patience could toil; and, without neglecting practical matter, we have willingly inserted interesting facts from many of the cognate sciences, believing that these are the things, "quæ quamvis medicum non faciant, aptiorem tamen ad medicinam reddunt."

Such have been the chief points at which we have aimed, —with what success it is for the profession to determine.

